# Kinder- und Jugendpsychiatrische Versorgung im Bereich Mental Health Rehabilitation in Österreich

**DOI:** 10.1007/s40211-022-00444-x

**Published:** 2022-12-19

**Authors:** Brigitta Lienbacher, Leonhard Thun-Hohenstein

**Affiliations:** 1 Psychosoziales Therapiezentrum, Bereich Kinder und Jugendliche, Kärnten, Villach, Österreich; 2 Paracelsus Medizinische Privatuniversität (PMU), Salzburg, Österreich

**Keywords:** Mental Health Rehabilitation, Kinder und Jugendliche, Kinder- und Jugendpsychiatrie, Versorgung, Mental Health Rehabilitation, Children and Adolescents, Child and Adolescent Psychiatry (CAP), CAP-Care

## Abstract

**Hintergrund:**

Mental Health-Rehabilitation für Kinder und Jugendliche wurde aufbauend auf einem Rehabilitationsplan von 2016 in Österreich ab 2018 zur Umsetzung gebracht. In der Zwischenzeit sind drei Rehabilitationszentren in 3 verschiedenen Versorgungszonen (Ost, Nord, Süd) errichtet und in Betrieb genommen worden.

**Methodik:**

Es wurde eine Anfrage bei der ÖGK um aktuelle Zahlen betreffend Aufnahme und Diagnosen durchgeführt sowie eine Stellungnahme der Leiterinnen der österreichischen Reha-Zentren herangezogen, um anhand dieser Daten und Fakten und im Lichte der vorangegangenen Planungen die Umsetzung der Mental Health Rehabilitation für Kinder und Jugendliche in Österreich zu dokumentieren und kritisch zu analysieren.

**Ergebnisse:**

In den drei Zentren wurden insgesamt 96 Behandlungsplätze eröffnet und in Betrieb genommen. Das vierte Zentrum soll 2022 in Betrieb gehen. Mit diesem wird die Bettenmessziffer im berechneten Bereich liegen, aber deutlich unter den Annahmen des Rehabilitationsplanes 2016. In Bezug auf die Strukturqualitätskriterien sind insbesondere die Konzeptualisierung und die Berechnung der Personalstrukturen als nicht ausreichend anzusehen. Die Umsetzung des Rehabilitationsplanes 2016 erfolgte nur teilweise, da nur MHR-Typ I Zentren errichtet wurden. Die Inanspruchnahme erscheint nach dreijähriger Betriebsdauer ausreichend zu sein, wobei das Diagnosespektrum aufzeigt, dass eher Kinder und Jugendliche mit akuten Problemen (Belastungsstörungen, depressive Episode) betreut wurden.

**Schlussfolgerung:**

Erfreulicherweise sind die beschriebenen Mental Health Rehabilitations-Zentren in Betrieb gegangen und wurden gut angenommen. Allerdings ist festzuhalten, dass das ursprüngliche Konzept verlassen bzw. reduziert wurde, dass die Strukturqualität und die Konzeptualisierung nochmals überdacht werden sollten und es dringend empfohlen wird, das ursprüngliche Konzept umzusetzen.

## Abstract (en)

**Background:**

Mental health rehabilitation for children and adolescents was implemented in Austria from 2018 onwards based on a rehabilitation plan from 2016. In the meantime, three rehabilitation centres have been set up and put into operation in 3 different supply zones (East, North, South).

**Methods:**

An inquiry was made to the ÖGK for up-to-date figures regarding admission and diagnoses and a statement by the heads of the Austrian rehabilitation centres was used to document and critically analyse the implementation of mental health rehabilitation for children and adolescents in Austria on the basis of these data and facts and in the light of the previous plans.

**Results:**

A total of 96 treatment places were opened and put into operation in the three centres. The fourth center is scheduled to go into operation in 2022. With this, the bed rate will be in the calculated range, but well below the assumptions of the rehabilitation plan 2016. With regard to the structural quality criteria, in particular, the conceptualisation and calculation of personnel structures are not to be regarded as sufficient. The implementation of the 2016 rehabilitation plan was only partial, as only MHR Type I centers were established. The use seems to be sufficient after three years of operation, whereby the diagnostic spectrum shows that children and adolescents with acute problems (stress disorders, depressive episode) were more likely to be cared for.

**Conclusion:**

Fortunately, the described mental health rehabilitation centers have gone into operation and have been well received. However, it should be noted that the original concept has been abandoned or reduced, that the structural quality and conceptualization should be reconsidered, and it is strongly recommended to implement the original concept.

## Einleitung

Rehabilitation schließt nach der allgemeinen Definition der Weltgesundheitsorganisation alle jene Maßnahmen ein, die darauf ausgerichtet sind, das Ausmaß von Einschränkung der Aktivitäten und Partizipation zu verringern, um eine gute soziale Integration zu erreichen. Rehabilitation zielt also nicht nur darauf ab, beeinträchtigte oder behinderte Personen zu unterstützen in ihrer Umgebung zurecht zu kommen, sondern auch Umgebungsfaktoren und die Gesellschaft so zu beeinflussen, dass deren soziale Integration erleichtert wird. Nach der ICF (Internationale Klassifikation der Funktionsfähigkeit, Behinderung und Gesundheit [1]) stellt Rehabilitation das personenbezogene multi- und interdisziplinäre Management von Beeinträchtigungen der Funktionsfähigkeit dar. Psychiatrische Rehabilitation im engeren Sinn umfasst alle jene Maßnahmen, die einen seelisch kranken Menschen über die Akutbehandlung hinaus durch umfassende Maßnahmen auf medizinisch, schulisch, beruflich und allgemeinen sozialem Gebiet in die Lage versetzen, seine Lebensform und Stellung, die ihm entspricht, zu finden bzw. wiederzuerlangen [2].

Nach einer anderen Definition [3] ist psychiatrische Rehabilitation die systematische Anwendung von Interventionen, die entwickelt wurden um Schädigungen (Impairment), Funktionseinschränkungen (Disabilities) und soziale Beeinträchtigung (Handicap) zu reduzieren. Das Ziel psychiatrischer Rehabilitation ist es daher sicherzustellen, dass Menschen mit psychischen Beeinträchtigungen die körperlichen, emotionalen und intellektuellen Fähigkeiten entwickeln können, um in ihrer Gemeinschaft zu leben, zu lernen und zu arbeiten. In der Definition der Deutschen Gesellschaft für Kinder- + Jugendpsychiatrie und Psychotherapie [4] wird Kinder- + Jugendpsychiatrische Rehabilitation als Psychosoziale Rehabilitation bezeichnet und damit eine inhaltlich etwas weitere Definition vorgenommen, die somit psychosomatische und sozialpädiatrische Problemfelder ebenfalls mit einschließt. Unter Psychosozialer Rehabilitation definiert die Leitlinie einerseits Hilfe zur Krankheitsverarbeitung und Bewältigung von Behinderung und andrerseits die Förderung der sozialen Kompetenz, der Reduktion krankheitsbedingter Handicaps, der Teilnahme am altersentsprechenden sozialen Leben inkl. Arbeit und Schule sowie der Verwirklichung des Rechtes auf ein selbstbestimmtes Leben. Rehabilitation soll nicht mehr die Herstellung bestmöglicher Gesundheit, sondern die Gewährleistung einer weitgehend normalen Teilhabe am Leben in der Gesellschaft zum Ziel haben [5].

Für die Kinder- + Jugendpsychiatrie in Österreich ist die Rehabilitation Bestandteil der Definition des Fachgebietes und damit auch fixer Bestandteil der Ausbildung auf diesem Fachgebiet [6, 7]. In der Leistungsorientierten Krankenhausfinanzierung (LKF) gab es unter der Kategorie Kinder- und Jugendpsychiatrie ein eigenes Leistungs- und Strukturqualitätsschema für rehabilitative Behandlung (KJNP‑R, MEL 7501) im Rahmen der Spitalsbehandlung und das somit die Phase Akut-Rehabilitation abdeckte (siehe auch Abb. 2). Durch den Wegfall dieser Kategorie im LKF ist nun keine eigene KJP-Rehabilitation für psychisch kranke Kinder und Jugendliche mehr vorgesehen – im Gegensatz zur Pädiatrie [8, 9]. Nach Vorarbeiten der Gesundheit Österreich (GÖG) mit Expert:innen aus der Pädiatrie und Kinder- und Jugendpsychiatrie [9] wurde im Rehabilitationsplanes 2016 [10] erstmalig eine Rehabilitation im Bereich Mental Health für Kinder und Jugendliche angedacht und mit der Kinder-Gesundheitsstrategie [11] als eigenes Gesundheitsziel (Ziel 18; p. 77) die Umsetzung beschlossen. Die angedachte Bettenmessziffer für Mental Health Rehabilitation für Kinder und Jugendliche zu diesem Zeitpunkt wird mit 110–220 angegeben [8] als eine Kombination aus kinder- und jugendpsychiatrischen und psychosomatisch-sozialpädiatrischen Betten. Die Vergabe erfolgte an private Gesundheitsträger. Die Eröffnung der ersten MH-Kinderreha erfolgte im März 2018 in Wildbad Einöd. Seither wurden noch 2 weitere Einrichtungen eröffnet, in Rohrbach/OÖ und Bad Erlach/NÖ; die Einrichtung in Wiesing/Tirol ist im Bau und soll im Herbst 2022 eröffnet werden.

### Definition der Versorgungsebene

Rehabilitation in der KJP hat zwei Versorgungsebenen, einmal in der Nachsorge einer Akutbehandlung im Krankenhaus und andrerseits im engeren Sinne der Rehabilitation, einer ambulanten und stationären Nachbehandlung oder tertiären oder quartären Prävention. Nach dem Rehabilitationsplan 2016 [10, p. 16] steht Rehabilitation „in ursächlichem und zeitlichem Zusammenhang mit der akutmedizinischen Versorgung“. Das heißt, es muss eine – von der Akutmedizin definierte – Rehabilitationsbedürftigkeit und -fähigkeit vorliegen und eine damit verbundene – möglichst positive – Rehabilitationsprognose möglich sein.

### Definition des Versorgungsauftrages

Der Versorgungsauftrag [10] umfasst die Wiederherstellung des Gesundheitszustandes des Versicherten und ihrer Angehörigen mit dem Ziel eine „Bestmögliche Wiederherstellung der Gesundheit im Sinne des bio-psycho-sozialen Krankheitsmodells (Restitutio ad Optimum) durch Einsatz eines interdisziplinären Rehabilitationsteams“ zu erreichen. Im Sinne des ICF-Modells [1, 10] sollen dabei „Schädigungen/Funktionsstörungen, Fähigkeitsstörungen und Beeinträchtigungen beseitigt, verbessert oder hintangehalten werden.“

### Bedarfsangaben zu Anzahl der beteiligten Berufsgruppen, Personen, geographische Region/Relation zu Bevölkerungszahlen

Im Rehabilitationsplan 2016 [10] wird das Gebiet der Kinder- und Jugendpsychiatrie und der Pädiatrie (i.e. Sinne Entwicklungs- und Sozialpädiatrie, ESP) unter dem Überbegriff Mental Health Rehabilitation zusammengefasst. Prinzipiell wird festgehalten, dass Mental Health Rehabilitation „möglichst wohnortnahe, ambulant oder teilstationär angesiedelt werden soll, um eine möglichst gute Rückbindung an die primären Umgebungsbedingungen und – um das Hauptziel – die Reintegration in Familie, Schule und/oder Arbeitsumfeld – bestmöglich zu gewährleisten“ ([10]; S. 121).

Laut einer nicht veröffentlichen Berechnung aus dem Jahre 2012 (Thun-Hohenstein L., Spiel G. persönliche Mitteilung) wurde anhand einer Umfrage an den österreichischen Abteilungen für KJP, und der Auswertung einer ambulanten und einer stationären Einrichtung der Versuch unternommen, den österreich-weiten Bettenbedarf für eine KJP-Rehabilitation zu erheben. Dabei wurde ein Bedarf von 96–135 Betten errechnet, der den damals, auf Österreich umgerechneten Daten aus der BRD entsprach. Der Hauptverband hat 2016 den Bettenbedarf mit 42 KJP ([10]; S. 157) und für ESP 68 berechnet und somit den 2010 von der Gesundheit Österreich [10] erarbeiteten Plan deutlich unterschritten. Für den Aufbau der Reha-Versorgung wurde Österreich in vier Versorgungszonen aufgeteilt: Versorgungszone Ost: Wien und Niederösterreich, Versorgungszone (VZ) Süd: Kärnten, Steiermark und das Burgenland, Versorgungszone (VZ) Nord: Oberösterreich und Salzburg und Versorgungszone West mit Tirol und Vorarlberg. Die für 2020 zu erreichende Bettenverteilung auf die Versorgungsregionen wurde wie folgt angegeben [9, S. 169]: VZ OST 46, VZ SÜD 24, VZ NORD 24 und VZ WEST 15, gesamt also 109 Plätze.

### Strukturqualitätskriterien/Mindeststandards

In Tab. 1 sind die im Rehabilitationsplan 2016 [10] gelisteten Indikationen aus dem ICD-10 und dem DC:0–5 [12] aufgeführt. Ebenfalls wurden entsprechende Kontraindikationen für den Mental Health Bereich formuliert: akute Selbst- oder Fremdgefährdung, akute psychische Störungen und für spezifische Störungsbilder (Essstörungen) für die in der Rehabilitationseinrichtung keine ausreichende Expertise vorgehalten werden kann. Für eine Aufnahme an einer KJP-Rehabilitation muss eine Erkrankung aus dem Kreis der Indikationen vorliegen, eine von der Akutbehandlungseinheit erstellte Rehab-Diagnose und -Prognose sowie die Einwilligung des Versicherungsträgers vorliegen.

**Table 1:** 

Allgemeine Indikationen nach ICD-10	F1: nicht-substanzgebundene Süchte
	F2: Schizophrenie, schizotype und wahnhafte Störungen in der Remissionsphase
	F3: Affektive Störungen
	F4: Neurotische, Belastungs- und somatoforme Störungen: v. a. F44, F45
	F5: Verhaltensauffälligkeiten mit körperlichen Störungen und Faktoren: F51, F54, F59: keine Essstörungen (F5) bzw. in der Stabilisierungsphase
	F7: Intelligenzstörung
	F6: Persönlichkeits- und Verhaltensstörungen
	F8: Entwicklungsstörungen (insbes. kombinierte E.; Autismus)
	F9: Verhaltens- und emotionale Störungen mit Beginn in der Kindheit und Jugend
Rehab-Spezifische Indikationen	*1. Aus der Gruppe der ICD-10:*
	F0: F07 organische Persönlichkeitsstörung
	F4: F44 dissoziative Störungen, F45 Somatoforme Störungen
	F5: F51 nicht-organische Schlafstörungen, F54 Psychosomatische Erkrankungen, F59 nicht näher bez. Verhaltensauffälligkeiten bei körperlichen Erkrankungen. Die Rehabilitation von Essstörungserkrankten sollte in spezialisierten Zentren erfolgen (s. a. Kontraindikationen)
	F7: verschiedene Formen
	F8: nur bei Vorliegen außergewöhnlicher Belastungen/weiterer psychiatrischer Symptome, F84 frühkindlicher Autismus
	F9: F90 Hyperaktivitäts- und Aufmerksamkeitsstörung, F93 emotionale Störung mit Trennungsangst, F95 vorübergehende Tic-Störung
	*2. Diagnosen nach dem Bereich der (DC 0–5)*^*a*^* Frühen Kindheit*
	*3. Pädiatrische Diagnosen:*
	Syndrome wie Morbus Down (Q91) oder Turner-Syndrom (Q92) und andere genetisch bedingte Erkrankungen mit psychiatrischen Symptomen
	Adipositas E66
	Chronisch pädiatrische Erkrankungen mit massiven Compliance-Problemen, Belastungsreaktionen u. Ä. (z. B. Diabetes, Asthma, chron. entzündliche Darmerkrankungen, kardiologische Erkrankungen, etc.)

^a^Lienbacher B., Reiter M. & Köstlinger-Jakob B. (2021) Brief der Leiterinnen der Mental Health K+J Einrichtungen an die ÖGK (persönliche Mitteilung)

Das „Medizinisches Leistungsprofil: Struktur‑, Prozess- und Ergebnisqualität in Vertragseinrichtungen für stationäre Rehabilitation von Kindern und Jugendlichen“ des Hauptverbandes der Sozialversicherungen umfasst eine Mindestbehandlungsmenge von durchschnittlich 150 min Therapie täglich oder bei einem Aufenthalt von geplanten 36 Tagen 3600 min Therapie, die zu 30 % psychologisch-psychotherapeutisch sein sowie 7 ärztliche Visiten beinhalten und ca. 30 % als Einzeltherapie angeboten werden müssen.

In Tab. 2 sind die Personalrichtwerte für die Mental Health Rehabilitation angeführt.

**Table 2:** 

Ärztlicher Dienst	Mind. 50 % FÄ für KJP oder KJH; weitere FÄ in Ausbildung oder AM	PKZ: 1:10 (Exkl. Ärztliche Leitung)
Ärztliche Leitung ist bis zu einer Bettenzahl von 49 ist mit einer halben PKZ einzurechnen	–	–
Psychologischer Dienst Klinische- und Gesundheitspsychologinnen mit Eintragung in die Liste des BM vorzugsweise mit Zusatzqualifikation oder Vorerfahrung im KiJuBereich	–	PKZ 1:15
Psychotherapeutischer Dienst Mit Eintragung in die Liste des BM vorzugsweise mit Zusatzqualifikation oder Vorerfahrung im KiJu-Bereich	–	PKZ 1:10
Pflegedienst DGKP mit Zusatzqualifikation KiJu- (was aus Mangel an Kolleginnen nicht möglich ist)	Die SP sind in die PKZ einzurechnen bis max. 50 %	PKZ 1:6
Gehobener medizinischer Dienst Physiotherapie/SPOWI/Logopädie/Ergotherapie	–	PKZ 1:10
Diätologie		PKZ 1:60
Sonstiges med. Personal: Med. Masseure	–	PKZ 1:45
Sonstiges Personal: Sozialarbeiterin Heilpädagoginnen Sozialpädagoginnen Musiktherapeutinnen	–	– PKZ 1:40 Wünschenswert! Wünschenswert! Wünschenswert!

Diese Arbeit versucht einen Überblick über die Umsetzung seit 2018 zu geben und anhand von Zahlen und Erfahrungen eine Diskussion über diese Versorgungsmaßnahme anzustoßen.

## Methode

Für eine, im Rahmen einer gemeinsamen Präsentation aller Mental Health K + J-Reha-Einrichtung beim ÖGKJP Kongress im Herbst 2021 vorgestellten Präsentation wurden aktuelle Daten erhoben und die jeweiligen Leiterinnen konnten ihre Erfahrungen austauschen. Verbesserungsvorschläge wurden in einem gemeinsamen Paper gesammelt und den Ansprechpartner: innen der ÖGK übergeben^1^. Weiteres konnten über eine Anfrage bei der Dachverband der Sozialversicherungsträger aktuelle Daten über Fallzahlen, Diagnosen und Altersgruppen erhoben werden.

## Ergebnisse

Status quo der Mental Health Reha Versorgung in Österreich: 2018 wurde Wildbad Einöd (Versorgungszone Süd) und Rohrbach (Versorgungszone Nord) mit jeweils 24 Betten, 2019 in Bad Erlach (Versorgungszone Ost) weitere 48 Betten eröffnet. Für 2022 wird die Eröffnung des Standortes Wiesing (Versorgungszone West) mit weiteren 17 Betten erwartet. Somit sind insgesamt 96 Betten verteilt über Zentral und Ostösterreich bereits in Betrieb, gesamt wären es 113 und liegen damit etwas über dem SOLL [10].

### Zahlen des Dachverbandes der Sozialversicherungen

**Figure 1:**
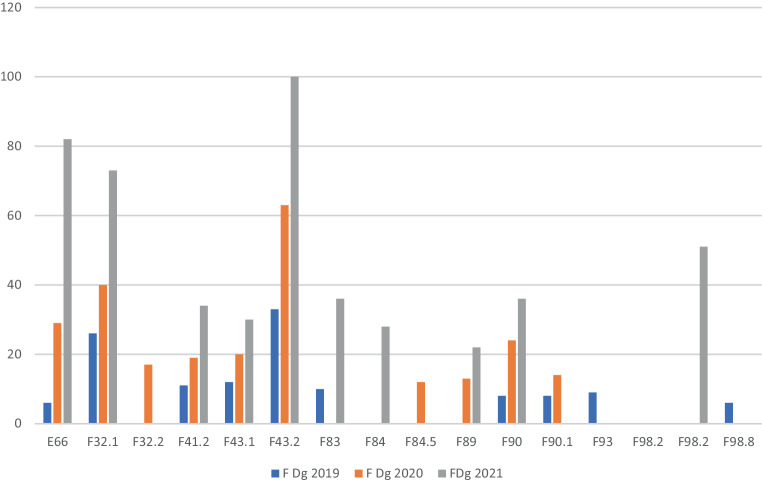

Nach Angaben der Dachverband der Sozialversicherungsträger wurden seit 2019 insgesamt 1745 Kinder und Jugendliche mit der Indikation Mental Health an den genannten Zentren behandelt. Von März 2020 bis Juni 2020 waren die Rehabilitationszentren aufgrund der COVID-19-Pandemie gesperrt. Im Durchrechnungszeitraum von 3 Jahren (2019–2021) ergibt das insgesamt 960 Plätze pro Jahr bei einer durchschnittlichen Auslastung von 60 %, wobei im Jahr 2021 996 Patient:innen behandelt wurden, was einer Auslastung von 100 % gleichkommt. Die häufigsten Diagnosen waren F43.2 (Anpassungsstörung, 23,5 %), F32.1 (depressive Episode, 16,7 %) und F90 (Hyperaktivitäts- und Aufmerksamkeitsstörung, 14,0 %). In Tab. 3 sind die Altersverteilung der Behandelten und in Abb. 1 die Häufigkeiten aller Diagnosen für die Jahre 2019–2021 aufgeschlüsselt.

**Table 3:** 

Alter	2019	2020	2021	Gesamt	%
*0–10*	76	166	447	689	39,48
*11–14*	56	116	186	358	20,52
*14+*	98	237	363	698	39,48
*Alle*	230	519	996	1745	100,0

Die wesentlichsten Forderungen der Stellungnahme der (ehemaligen) Leiterinnen der Mental-Health-Rehab-Einrichtungen Österreichs^2^ sind in Tab. 4 aufgelistet.

**Table 4:** 

Themenkomplex	Anforderung	Ist-Zustand	Erforderliche Änderungen
*Kleine Kinder* m. erhöhtem Förderbedarf	Mehr Einzelstunden, insgesamt weniger Therapieminuten	Die Therapieminuten tgl. und insgesamt sind für alle Altersgruppen gleich	Anpassung des LP an die unterschiedlichen Bedürfnisse der Altersgruppen: konkret erhöhen der Einzelminuten, Flexibilisierung der tgl. Minutenanzahl; Anrechnung von Heil- und Sonderpädagogik
*Betreuung in der therapiefreien Zeit*	Professionelle Betreuung auch außerhalb der Therapiezeiten	Die Betreuung der Kinder- und Jugendlichen außerhalb der Therapiezeit ist nicht geregelt und führt nicht selten zu einer Überforderung der Eltern/Begleitpersonen	Verpflichtende Aufnahme von Sozial‑, Sonder- und Heilpädagogik in eine eigene Personalkennzahl
*Schüler:innen*	Schulzeit ins Behandlungskonzept mit einbeziehen	Bei Schulbesuch Überforderung die tägliche Minutenzahl erreichen zu müssen	Reduktion bzw. Flexibilisierung der Minutenzahlen bei Schüler:innen
*Ferienzeiten*	Betreuungsnotwendigkeit höher	In der Ferienzeit keine ausreichende Betreuung außerhalb der Therapiezeit gewährleistet	Ausbau der Sozialpädagogischen Betreuung (Milieutherapie, Alltags- und Sozialtraining, Tagesstruktur Berufsorientierung) und Verschränkung mit pflegerischer Tätigkeit (Anpassung Tätigkeitsprofil Pflege)
*Begleitpersonen*	Sind für die therapeutische Arbeit mit jüngeren oder beeinträchtigten Kindern und Jugendlichen notwendig	Fehlende einheitliche Regelung zur Freistellung der Eltern	Verbindliche und einheitliche Urlaubs resp. Freistellungsregelung für Begleitpersonen
*Therapeutische Heimfahrten*	Erprobung der erarbeiteten therapeutischen Maßnahmen zu Hause	WE-Ausgänge über 2 Nächte erlaubt	Flexible, der Lebens- und Behandlungssituation angepasste Regelung
*Elternarbeit*	Fachlich und organisatorisch (gesetzlich) notwendig, dass Eltern mitarbeiten	Muss zurzeit vom Therapiekontingent der Kinder und Jugendlichen abgezogen werden	Ergänzung des Leistungsprofil um Leistungen für Eltern und Angehörige
*Vorzeitige Abbrüche*	Hohe Anforderung an Eigenmotivation	Häufig Patient:innen ohne Motivation	Regelung der finanziellen Gebarung bei vorzeitigen Abbrüchen
*Besprechungs- und Vernetzungszeiten*	Aufgrund der oft komplexen Lebenssituationen und der Dezentralität der Reha-Einrichtung hohes Maß an Besprechungszeit und Vernetzung nötig	Im LP nicht ausreichend berücksichtigt	Berücksichtigung im Leistungsprofil und in der Personalplanung zu berücksichtigen

^a^Lienbacher B., Reiter M. & Köstlinger-Jakob B. (2021) Brief der Leiterinnen der Mental Health K+J Einrichtungen an die ÖGK (persönliche Mitteilung)

## Diskussion

Trotz schwieriger Rahmenbedingungen war es Dank des Pioniergeistes der sehr motivierten multiprofessionellen Teams möglich, für die Kinder- und Jugendlichen hilfreiche Rehabilitations-Strukturen aufzubauen, die zumindest für die Dauer des Aufenthaltes durchwegs zu einer Verbesserung der jeweiligen Symptomatik geführt haben [13]. Vor allem während der Corona-bedingten Einschränkungen konnte eine Ruheoase geschaffen werden, in der die Jugendlichen in einem geschützten Rahmen die Möglichkeit hatten, mit Gleichaltrigen in Kontakt zu sein, die Schule zu besuchen und die notwendige Behandlung zu bekommen. Befragt, was am meisten geholfen hat, antwortete die Mehrheit der betreuten Jugendlichen: die Tagesstruktur und die sozialen Beziehungen zu den anderen Jugendlichen sowie die psychotherapeutischen Einzelgespräche [13]. Als weitere positive Einflussfaktoren wurden in der erwähnten Studie von den Jugendlichen der Abstand von Zuhause (i.S. Abstand von der belastenden Situation), Zeit für sich haben, ich darf ich selbst sein („ich bin ok“), das Experimentieren, das Gemeinschaftsgefühl und die ständige Anwesenheit erwachsener Personen, um ein Gespräch führen zu können, angeführt. Wesentlich ist offensichtlich der multiprofessionelle Therapiemix, der mentale, soziale, strukturelle und körperliche Faktoren gleichermaßen berücksichtigt.

Im Hinblick auf die Planung der Mental Health Rehabilitation aus dem Jahr 2016 wurde das vom Hauptverband der Sozialversicherungen selbst herausgegebene Konzept praktisch vollständig verlassen. Wurden in diesem ursprünglichen Konzept vier sehr unterschiedliche Formen der MH-Reha empfohlen: wohnortnahe und ambulante Reha, dezentrale Reha-Zentren und kooperativ/integrative Formen mit Pädiatrie und Kinder- und Jugendhilfe, so wurden im Endeffekt nur vier dezentrale Reha-Zentren gemeinsam mit der Pädiatrie (MHR Typ I n. Rehabilitationsplan 2016) errichtet. Weiters wurde die theoretische Bedarfszahl an Mental-Reha-Plätzen deutlich unterschritten, wenn man nur für die Kinder- und Jugendpsychiatrie rechnet. Gemeinsam mit den pädiatrisch-psychosomatischen Plätzen liegt die Umsetzung in der Mitte des berechneten Solls, aber deutlich unter dem von der Gesundheit Österreich GmbH (GÖG) berechneten Solls von 220 Plätzen [8].

Die geographischen Verteilung der Zentren bedeutet für viele Familien, dass diese Zentren weitab vom Lebensmittelpunkt liegen und damit auch eine Rückbindung in den Alltag und eine entsprechende Nachbetreuung sehr schwierig organisierbar sind. So liegen Lienz (190,3 km) und Bad Gleichenberg (182,3 km) beide mehr als 2,5 h per Auto vom zuständigen Reha-Zentrum der Versorgungsregion entfernt (für die zuweisenden KJP-Abteilungen noch viel weiter!), öffentlich sind die Zentren noch viel schlechter erreichbar. Da bei der Behandlung von psychisch kranken Kindern und Jugendlichen die Mitbetreuung und teilweise Behandlung der Eltern in der Regel zwingend notwendig ist, ist das eine dramatische Erschwernis – ganz abgesehen von einer Nachbetreuung (die im Reha-Plan vor Ort gar nicht vorgesehen ist) und dem Aufwand, den Eltern dafür betreiben müssten!

Das Leistungsprofil der ÖGK regelt in Anlehnung an die Leistungsprofile für Psychiatrische Reha der PVA wichtige Rahmenbedingungen wie Therapieausmaß und Personalausstattung. Auf die unterschiedlichen Bedürfnisse von Kinder und Jugendlichen wurde kaum Rücksicht genommen, wie in Tab. 4 dokumentiert. In dieser Tabelle sind einige wesentliche Forderungen mit entsprechender Begründung dargestellt. Es handelt sich um bisher nicht ausreichend im Leistungsprofil abgebildete Angebote bzw. Strukturqualitätskriterien, die für eine standardgemäße Betreuung psychisch kranker Kinder und Jugendlicher und ihrer Angehörigen aber notwendig sind. Im Rehabilitationsplan 2016 wurden für vier verschiedenen Rehabilitations-Strukturen differenzierte Strukturqualitätskriterien vorgelegt, die in deutlich abgeänderter Form ins Leistungsprofil der neuen Reha-Zentren eingebracht wurde. Legt man die unterschiedlichen Störungsbilder, Altersgruppen, individuellen Bedürfnisse und Familienkonstellationen der PatientInnen einer Planung zugrunde, so ergibt sich ein hochkomplexes Anforderungsprofil, das mit der vorhandenen Personalstruktur nur schwer ausreichend versorgt werden kann. Die oben angeführte Sachlage (Komplexität der Störungsbilder, Altersgruppen, Bedürfnisse etc.) machen neben einem gut strukturierten therapeutischen Grundkonzept auch einen bedürfnisorientierten Planungsspielraum erforderlich. Diese Möglichkeit zur individuellen Anpassung des Therapieplanes ist derzeit im Leistungsprofil ebenfalls nicht ausreichend abgebildet. Der organisatorische und betreuerische Zusatzaufwand, der sich dadurch ergibt, wurde in den Personalkennzahlen nicht berücksichtigt und schmälert dadurch die Ressourcen, die für die patientenbezogene Arbeit zur Verfügung stehen. Es wurde verabsäumt, die notwendige professionelle Betreuung der Kinder und Jugendlichen in den therapiefreien Zeiten als integrativen Bestandteil des Rehabilitationsaufenthaltes zu werten. SozialpädagogInnen, Elementarpädagog:innen und Sonder‑/Heilpädagog:innen wurden als „wünschenswert“ in andere Berufsgruppen eingerechnet. Das Schaffen von klaren Tagesstrukturen und geregelten Alltagsabläufen trägt jedoch wesentlich zum Therapieerfolg bei und bedarf einer professionellen Herangehensweise durch die oben genannten Berufsgruppen. Eine weitere Schwierigkeit ergibt sich aus der Notwendigkeit, die PatientInnen für die Behandlung in Altersgruppen zu unterteilen. Unterschiedliche Gruppengrößen und inhaltliche Anforderungen erhöhen der Aufwand für die Planung und den Personalaufwand erheblich. Besonders für die Betreuung von unter 3‑jährigen Kindern wird eine eigene Infrastruktur und hochspezialisiertes Personal benötigt. In dieser Zielgruppe sind in erster Linie pädiatrisch-psychosomatische Störungsbilder zu finden, aber auch psychosoziale Indikationen wie psychisch belastete Eltern und Eltern-Kind-Interaktionsstörungen. Daher ist dafür ein eigenes Konzept notwendig, das Elternteil und Kind gleichermaßen im Leistungsprofil berücksichtigt, wie es bei der FOR (familienorientierte REHA) der Fall ist. Da es keine einheitliche Regelung gibt, ob Eltern Urlaub nehmen können oder vom Dienstgeber freigestellt werden, limitiert sich diese PatientInnengruppe automatisch und betroffene Familien können ein stationäres Reha-Angebot nicht nutzen.

Dennoch wurden die Mental Health-Reha-Zentren recht gut angenommen, da 2021 die Auslastung auf nahezu 100 % gestiegen ist. Die Altersgruppen sind ziemlich harmonisch drittelmäßig verteilt, allerdings ist es nicht möglich den Anteil von Kindern im Vorschulalter oder kleiner abzuschätzen. Aus den vorhandenen Diagnosen ist dies auch nicht ablesbar, da es keine Diagnosen aus dem Klassifikationsschema 0–3 gibt, auch kann aus den übermittelten Zahlen auch nicht die Frequenz einer Eltern-Kind-Aufnahme herausgelesen werden. Die Diagnosen verteilen sich über das gesamte Indikationsspektrum mit einem Schwerpunkt bei Anpassungsstörungen und depressiven Episoden, an dritter Stelle ist eine pädiatrische Diagnose mit der Adipositas (E66). In einer eigenen Berechnung [14] wurden die Reha-Indikationen anhand des stationären Klientels einer österreichischen UK f KJP (*n* = 680) über drei Jahre berechnet. Davon wurde bei 13,5 % die Indikation zu einer Rehabilitation gestellt und 3 größere Gruppen identifiziert: psychiatrische Diagnosen im engeren Sinne (46 %), Persönlicheitsentwicklungsstörungen/externalisierende Störungen (43 %) und die Gruppe der Suchterkrankten (inkl. Essstörungspatient:innen mit 11 %). Die vom Dachverband der Sozialversicherungsträger erhobenen Zahlen zeigen, dass in der Gruppe psychiatrische Erkrankungen im engeren Sinne die Diagnosen Affektive Störungen und Belastungsstörungen überwiegen, bestimmte Gruppen gar nicht versorgt werden (psychotische Erkrankungen, Essstörungen), aber die Gruppe externalisierende Störungen dafür überproportional vertreten ist. Dafür finden sich auch pädiatrische Störungen (Adipositas E66). Die basale Indikationsstellung zur Rehabilitation lässt sich anhand der Diagnosen nur erahnen, wenngleich das Überwiegen der Anpassungsstörung (F43.1) und der depressiven Episode (F32.12) und das Fehlen bestimmter schwerer psychiatrischer Diagnosen eher auf akut belastete Kinder und Jugendliche hinweist und nicht so sehr auf ein klassisches Rehabilitationsklientel. Angesichts der dramatischen KJP-Unterversorgung im Akutbereich in Österreich verwundert das nicht weiter.

**Figure 2:**
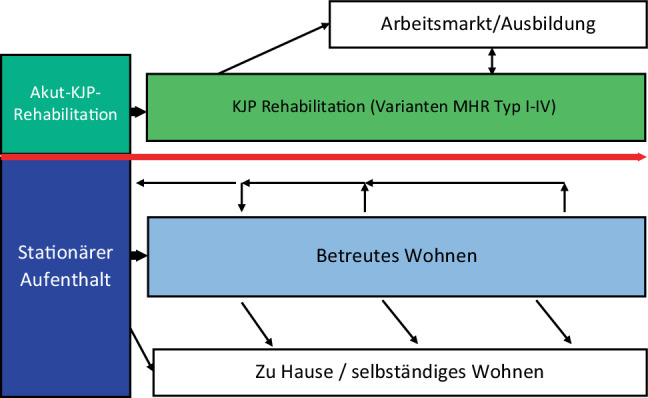

Abschließend soll noch festgehalten werden, dass gleichzeitig mit der Schaffung der Mental Health Rehabilitation die Leistung „KJP-Rehabilitation (LKF MEL 7501)“ im Leistungsorientierten Krankenhausfinanzierungssystem und somit die Möglichkeit, unmittelbar im Anschluss an die stationäre Behandlung eine Akutrehabilitation anbieten und verrechnen zu können, gestrichen wurde. In einem umfassenden Rehabilitationskonzept ist aber die Akutrehabilitation integraler Bestandteil einer standardgemäßen Versorgung (siehe Abb. 2.).

Der Rehab-Plan muss an jeder Stelle des Prozesses einen Umstieg in die primäre Lebenswelt ermöglichen, aber bei Versagen auch einen – für den Zustand des/r Kindes/Jugendlichen passenden – Wiedereinstieg in die Betreuung ermöglichen. Die Phasenstruktur der Psychiatrischen Rehabilitation wurde in Anlehnung an das Konzept der Neurorehabilitation bei Kindern und Jugendlichen [9] und der Versorgungsstruktur der Kinder- + Jugendpsychiatrie formuliert [7]. Aus diesem Grunde erscheint die Umsetzung des ursprünglichen Reha-Planes 2016 dringend nötig, um eine stufenweise und möglichst wohnortnahe Rehabilitation zu ermöglichen. Dies würde die Motivation der Betroffenen wesentlich steigern, da die Anfahrtszeiten schon eine hohe Anforderung an die Betroffenen darstellen und auch das Heimwehproblem bei einem doch nicht geringen Prozentsatz die Motivation der Patient: innen beeinträchtigt, war doch eine Eigenmotivation zu Beginn des Aufenthaltes nur bei 58 % vorhanden [13].

## Zusammenfassung

Unsere Erhebung und Darstellung zeigen, dass die Mental Health-Rehab-Zentren trotz erschwerter Bedingungen gut angenommen werden und drei Jahren nach Start eine hohe Auslastung aufweisen. Die diagnostische Bandbreite ist entsprechend, lediglich zeigten sich Hinweise darauf, dass in den Reha-Zentren eher Patientinnen aus der Akut-Psychiatrie zur Behandlung kommen. Gleichzeitig ist festzuhalten, dass das Konzept nachgeschärft gehört und die Strukturqualitätskriterien dringend adaptiert werden müssen, um eine standardgemäße Betreuung der Kinder und Jugendlichen und ihrer Angehörigen zu ermöglichen. Dringend empfohlen wird das ursprüngliche Konzept des Hauptverbandes der Sozialversicherungen [9] zur Umsetzung zu bringen und zusätzlich die Effektivität dieses Ansatzes und seiner Umsetzung zu evaluieren, da diese offenbar nicht bei allen Indikationen gegeben ist und für den Mental Health Bereich noch kaum aussagekräftige Studien vorliegen [15].

## Footnotes

1 Lienbacher B., Reiter M. & Köstlinger-Jakob B. (2021) Brief der Leiterinnen der Mental Health K+J Einrichtungen an die ÖGK (persönliche Mitteilung)

2 Lienbacher B., Reiter M. & Köstlinger-Jakob B. (2021) Brief der Leiterinnen der Mental Health K+J Einrichtungen an die ÖGK (persönliche Mitteilung)
